# Risk of tick-borne pathogen spillover into urban yards in New York City

**DOI:** 10.1186/s13071-022-05416-2

**Published:** 2022-08-10

**Authors:** Nichar Gregory, Maria P. Fernandez, Maria Diuk-Wasser

**Affiliations:** 1 Department of Ecology, Evolution and Environmental Biology, Columbia University, New York, NY USA; 2 Earth Institute, Columbia University, New York, NY USA; 3 Paul G. Allen School for Global Health, Washington State University, Pullman, WA USA

**Keywords:** *Ixodes*, *Haemaphysalis*, *Amblyomma*, Urban tick-borne disease, Landscape metrics

## Abstract

**Background:**

The incidence of tick-borne disease has increased dramatically in recent decades, with urban areas increasingly recognized as high-risk environments for exposure to infected ticks. Green spaces may play a key role in facilitating the invasion of ticks, hosts and pathogens into residential areas, particularly where they connect residential yards with larger natural areas (e.g. parks). However, the factors mediating tick distribution across heterogeneous urban landscapes remain poorly characterized.

**Methods:**

Using generalized linear models in a multimodel inference framework, we determined the residential yard- and local landscape-level features associated with the presence of three tick species of current and growing public health importance in residential yards across Staten Island, a borough of New York City, in the state of New York, USA.

**Results:**

The amount and configuration of canopy cover immediately surrounding residential yards was found to strongly predict the presence of *Ixodes scapularis* and *Amblyomma americanum*, but not that of *Haemaphysalis longicornis*. Within yards, we found a protective effect of fencing against *I. scapularis* and *A.* americanum, but not against *H. longicornis*. For all species, the presence of log and brush piles strongly increased the odds of finding ticks in yards.

**Conclusions:**

The results highlight a considerable risk of tick exposure in residential yards in Staten Island and identify both yard- and landscape-level features associated with their distribution. In particular, the significance of log and brush piles for all three species supports recommendations for yard management as a means of reducing contact with ticks.

**Graphical Abstract:**

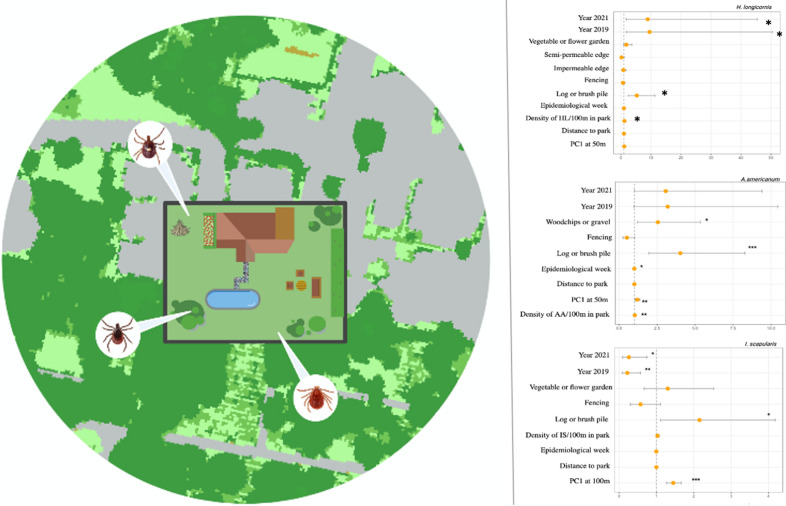

**Supplementary Information:**

The online version contains supplementary material available at 10.1186/s13071-022-05416-2.

## Background

Within the past two decades, reported cases of tick-borne disease (TBD) in humans have increased by more than twofold in the USA [1], with over 20 recognized human illnesses associated with ticks nationally [2]. In the northeastern USA, Lyme disease, caused predominantly by the bacterium *Borrelia burgdorferi* sensu stricto [3] accounts for the majority of disease burden [4], and its expansion has been associated with the geographic spread of the primary vector, *Ixodes scapularis* [5–10]*. Ixodes scapularis* is also a vector of multiple other pathogens of concern, including *Babesia microti *[11],* Anaplasma phagocytophilum *[12], *Borrelia miyamotoi* [13] and Powassan virus [14], which are also spreading throughout the USA [15, 16]. More recently, the lone star tick (*Amblyomma americanum*), which until recently has been considered a nuisance species and is most abundant in southern USA, has been spreading northward [17]. This species is associated with the transmission of *Ehrlichia chaffeensis* and *Ehrlichia ewingii,* agents of human granulocytic ehrlichiosis [18, 19], *Rickettsia rickettsii* Wolbach, the agent of Rocky Mountain spotted fever, and *Francisella tularensis* McCoy, the agent of tularemia [20]. In some emerging areas in the northeastern USA, *A. americanum* has surpassed *I. scapularis* as the most commonly reported human-biting tick [21]. Similar range expansions have been observed with the Gulf Coast tick (*Amblyomma maculatum*), which was historically limited to a narrow coastal band in southeastern USA [22] and is now found in several inland states [23, 24] and, most recently, in highly urbanized New York City [25, 26]. Gulf coast ticks are the primary vector for *Rickettsia parkeri*, which causes American boutonneuse fever in humans [23]. The Asian longhorned tick (*Haemaphysalis longicornis*), a recent invader of the USA and recorded in the northeastern USA for the first time in 2017 [27–30], has been reported parasitizing humans [31, 32]. In its native range, this species is a vector for a suite of human pathogens, including severe fever with thrombocytopenia syndrome virus (SFTSV; [33]) and Japanese spotted fever [34]. Longhorned ticks have also been demonstrated to be competent for Bourbon virus [35] and Heartland bandavirus, a virus genetically similar to SFTSV, with both primarily vectored by the lone star tick [36].

The absence of human vaccines for endemic and emerging tick-borne pathogens in the USA has led to a focus on individual preventative measures to reduce tick encounters [37]. This includes recommendations for altering two key components of infection risk: (i) the acarological hazard (defined as the density of pathogen-infected nymphal ticks [DIN]) and (ii) human exposure behaviors [38]. Several studies have identified a positive association between DIN and human incidence [39–42], although the strength of this association varies spatially [43]. Recreational areas have been identified as high risk environments for exposure to infected ticks [44–48], particularly at woodland-lawn ecotones where tick densities are often the greatest [44, 49]. In suburban residential yards, frequently cited risk factors for the acarological hazard include proximity to woodland, lack of fencing, log and brush piles in the yard, bird-feeders and pet ownership [42, 44, 50–52], all of which may enhance the number of hosts and tick off-host survival.

The acarological hazard is determined by complex interplay between the local abiotic conditions conducive to tick persistence [53, 54] and the presence and abundance of hosts that support tick populations and pathogen persistence [55–57]. As ixodid ticks spend the majority of their lives off-host, local abiotic conditions are critical for determining local tick survival, development and activity [58, 59]**.** Thermal and desiccation tolerance of different tick species determines their habitat niche [60, 61], in turn impacting their host niche breadth through mediating exposure to hosts [62]. The high sensitivity of *I. scapulari*s to desiccation means that this species is typically associated with forests where leaf litter and high canopy cover drive high humidity conditions conducive to host-seeking behavior and tick survival [63]. In contrast, other tick vector species, such as *A. americanum*, *Dermacentor variabilis* and *H. longicornis* have wider tolerances for microhabitat conditions and can occupy grassland habitats in addition to forested sites, as well as ecotonal habitats subject to human disturbance [64–68].

Urban areas are increasingly recognized as frontiers for TBD expansion within endemic regions [46, 69], but the risk factors for acquiring TBD in these areas remain largely unknown. Urban landscapes are unique in terms of their extreme levels of habitat fragmentation, warmer and drier microclimates [70] and reduced wildlife diversity [71] compared to surrounding natural areas, although green spaces (e.g. urban parks) may mitigate these conditions by acting as wildlife refugia or dispersal corridors. As ixodid tick long-distance dispersal is mediated entirely by hosts [72], the community of hosts and the impact of landscape on host behavior can profoundly shape tick distribution [46, 73]. Tick populations in urban parks and natural areas thus form metapopulations, i.e. connected subpopulations that are reliant on other subpopulations for persistence [74, 75], and the extent to which patches are functionally connected by the movement of hosts through suitable habitats determines whether populations can persist in these patches. Abiotic and yard-specific features acting as attractants or barriers then determine whether hosts can transport ticks into yards. Deer, in particular, are key agents for structuring tick populations in urban parks and surrounding neighborhoods [46] due to their roles as reproductive hosts for adult ticks [76]. Differences in the host and habitat associations of different tick species may thus produce relationships between acarological hazard and habitat that varies across spatial scales [42, 51, 77, 78], necessitating a combined focal and landscape-level approach.

In this study, we seek to elucidate the drivers of tick distribution across an urban landscape focusing on Staten Island (SI), New York City (NYC), a newly emerging area for TBD. In our previous study of tick populations in NYC parks, we found the highest tick burden to be in SI parks [46]. We take a multi-scale approach to investigate the associations between landscape heterogeneity and yard features, and the occurrence of three ticks of public health concern: *I. scapularis*,* A. americanum* and *H. longicornis*. We hypothesize that the risk of TBD is hierarchically structured, depending primarily on the yard’s connectivity to natural areas at the ‘ecological neighborhood’ scale, and secondarily on the yard’s habitat suitability for ticks and potential permeability to hosts [79].

## Methods

### Study design and sites

Staten Island is one of five boroughs of NYC in the US state of New York. It spans 156 km^2^ and is the least populated borough of NYC, with 468,730 individuals [80, 81]. The island is composed of heterogeneous neighborhoods, with variable housing structure types and demographic and socioeconomic composition; 18% of the total area is covered by urban parks [80]. SI presents a network of discrete patches of urban parks of different sizes, distributed across a range of housing development of low, medium and high intensity, representing varying levels of connectivity to host movement. The rate of locally acquired Lyme disease cases increased from 4 to 25 per 100,000 residents between 2000 and 2016 [82].

**Fig. 1:**
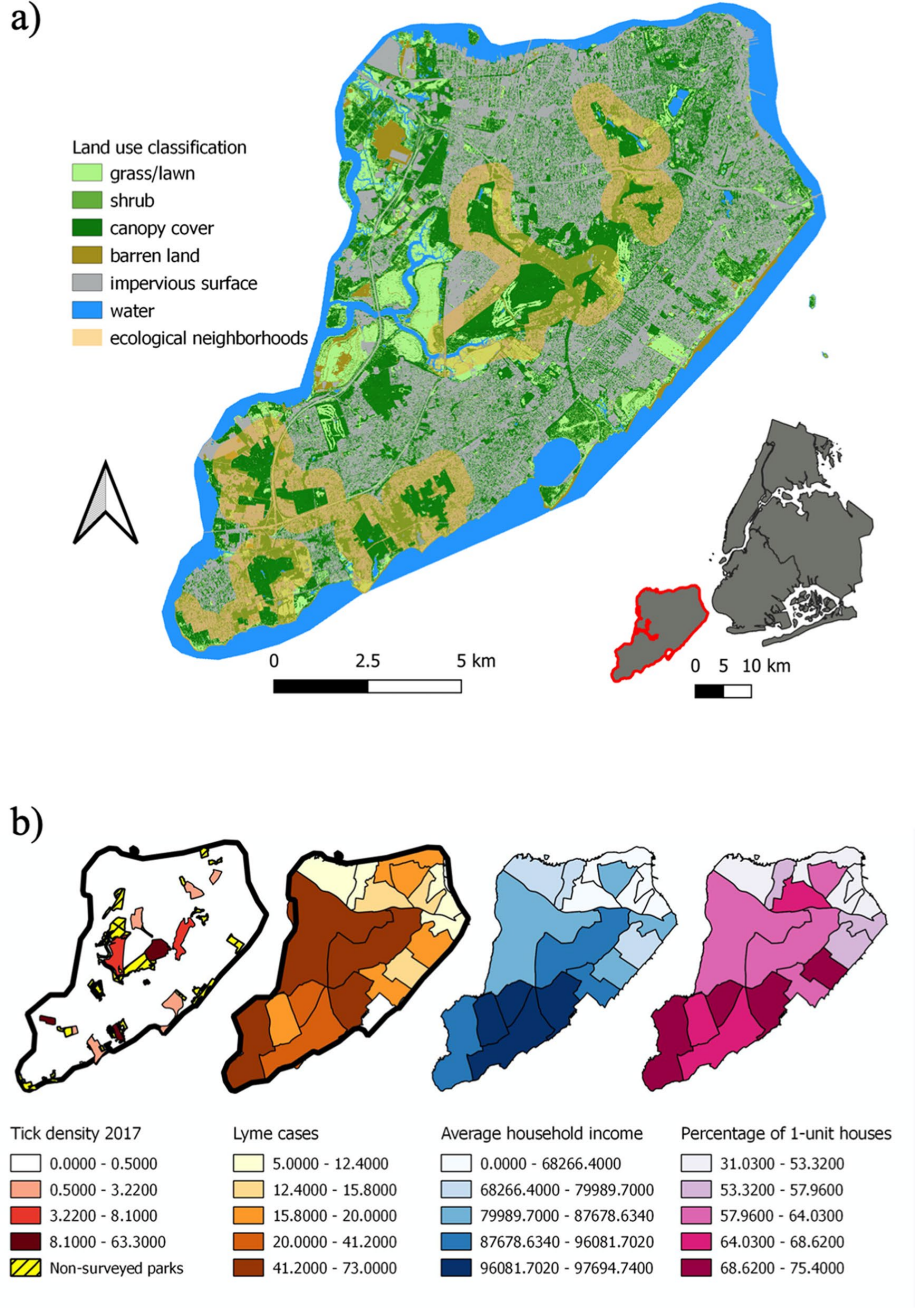
Location of sampling sites in Staten Island, New York City (**a**) and neighborhood characteristics (**b**).** a** Buffers, shown in yellow, denote ecological neighborhoods, defined as areas within 500 m of parks, within which houses were primarily sampled.** b** Demographic and eco-epidemiological information which highlights the variation in tick-borne disease risk and associated risk factors for tick-borne disease across the study area. Tick density refers to the total number of nymphs per 100 m. Lyme cases are the total number of cases reported from each neighborhood from 2010 to 2016. Average household income is given in USD

In this study, we use the concept of ‘ecological neighborhoods’ [79, 83], which describes an area within which an ecological process of interest occurs, taking into account the time scale appropriate to that process and the focal organism's activity or influence during that period [79, 84, 85]. Across SI, we defined ecological neighborhoods as areas encompassing a core park and the surrounding residential areas within 500 m from the park edges (Fig. 1a); this is a distance consistent with deer home-range size (43-158 ha or a radius of 370–700 m) [86], white-footed mice average dispersal distance [87] and the estimated human walkable distance used in urban design (400 m) [88]. The neighborhoods were selected to cover a range of urbanization levels on SI (Fig. 1b) and although sampling occurred across the entire island, it was concentrated in the mid- and south-mid sections due to a greater availability of park-adjacent houses.

Within these ecological neighborhoods residents were actively recruited using a random cluster sampling strategy. We randomly selected starting points along each neighborhood cluster using the points-to-polygon function in QGIS, a geographic information system application, which creates a points layer of randomly placed points within the input polygon, i.e. ecological neighborhood. From each point, we followed a line transect until 10–15 houses were recruited per cluster. We also passively recruited residents through a combination of targeted advertising in newspapers and online platforms. House visits were conducted from May through July 2018, 2019 and 2021. Sampling did not occur in 2020 due to the coronavirus disease pandemic. At each property, we recorded yard-level features that could be associated with the presence of ticks by attracting or deterring hosts or modifying the microhabitat for the ticks, such as the presence of log or brush piles, woodchips or gravel at the edge of the property, vegetable or flower gardens and bird feeders. We also collected data on fencing around yards, recording fence type, (aluminum, chain link, wood picket, full panel, farm fence and other), whether yards were completely or partially fenced and estimated fence heights.

Ticks were sampled from April to July in 2018, 2019 and 2021 by dragging a 1 × 1-m corduroy cloth at ground level along vegetation within each property and at each property edge. Each property was sampled once in any given year, and the total area sampled ranged from 10 to 400 m, which was proportional to the size of each residential yard. The transects dragged were located at the edges of the property and around the house (Additional file 1: Figure S1). At 10-m intervals, ticks were counted and collected into 1.5-ml snap-cap microcentrifuge tubes containing 70% ethanol [89, 90]. Ticks were identified to species using established keys [91, 92].

### Land cover and Landscape-level metric calculation

The land cover and landscape-level features surrounding each residential yard were characterized. We combined a land cover product which uses 2017 4-band ortho-imagery to classify land cover at a 60-cm resolution (www.earthdefine.com/landcover), with a 1-m Canopy Height Model (CHM) dataset, which uses light detection and ranging (LiDAR) data to estimate the vegetation height. The resulting land cover dataset encompassed seven land classes: grass (0–1 ft), shrub (< 6 ft), low canopy (≤ 60 ft), high canopy (> 60 ft), bare soil, water and impervious surface. Low and high canopy classes were designated using the median tree height derived from the CHM as a cut-off point. We created buffers of 25-, 50-, 100- and 200-m radii around each residential property sampled and calculated the area and proportion of each land cover type, excluding water bodies, within each radius.

We used the “landscapemetrics” package in R [93], which uses a drop-in replacement for FRAGSTATS [94], to extract 16 class-level metrics representing the spatial distribution and pattern of both canopy classes combined (as the focal class; Table 1), including shape, area, edge and aggregation metric categories. We scaled and centered all class-level landscape metrics covariates for analysis and tested these and the area of three land cover categories (grass, canopy and impervious surface) for collinearity in predictor variables using the corrplot function [95]. Due to high correlation among variables at both the landscape and class scales, we performed standardized principal component analysis (‘PCA’ function, FactoMineR package [96]) to reduce the dimensionality of the dataset. The Varimax rotation method was used to derive orthogonal principal components, and the first two dimensions were used as variables in the statistical model.

**Table 1:** Class metrics used to describe patterns of canopy cover (focal class, combining high and low canopy cover) around residential yards

Category	Acronym	Metric name	Description
Aggregation	COHESION	Patch cohesion index	Connectedness of patches
	ENN_MN	Mean of Euclidean nearest-neighbor distance	Mean edge to edge distance to the nearest neighboring patch of the same type
	NP	Number of patches	Number of patches
	CLUMPY	Clumpiness index	Proportional deviation of the proportion of like adjacencies involving the focal class from that expected under a spatially random distribution
	nLSI	Normalized landscape shape index	Ratio of the actual edge length of focal class in relation to the hypothetical range of possible edge lengths of the focal class (min/max)
	AI	Aggregation index	Percentage of neighboring pixel, being the same land cover class, based on single-count method
	IJI	Interspersion and juxtaposition index	Measure of evenness of patch adjacencies, equals 100 for even and approaches 0 for uneven adjacencies
	MESH	Effective mesh size	Relative measure of patch structure based on probability that two randomly chosen points will be located in same patch
Area and edge	TE	Total edge	Total length (m) of all edges between focal class and all other classes
	ED	Edge density	Sum of all edges of focal class in relation to landscape area
	LPI	Largest patch index	Percentage of landscape covered by corresponding largest patch of each class
	GYRATE_MN	Mean radius of gyration	Mean distance from each cell to the patch centroid
Shape	CONTIG_MN	Mean of contiguity index	Spatial connectedness of cells in patches
Core area	TCA	Total core area	Sum of all core areas of all patches belonging to focal class
	CPLAND	Core area percentage of landscape	Percentage of core area of focal class in relation to the total landscape area
	NDCA	Number of disjunct core areas	Number of cells of focal class without neighbors with a different value other than itself

### Edge classification

To quantify the ‘permeability’ of a yard to hosts, we created a vegetation contrast raster layer using the CHM layer by estimating the height difference between any given pixel and its surrounding eight neighboring pixels. The values of this layer were classified in four categories: “no edge”; “1- to 3-m difference in vegetation type”; “3- to 9-m difference in vegetation type”; and “ > 10 m difference in vegetation type” and “edge between vegetation and non-vegetated surface (e.g. impervious surface, barren land, water)”. To characterize the type of edge in each property we generated an edge index by creating 1-m buffers around the property boundary and extracting the area of land cover classes in each, the area of each type of category of the vegetation contrast layer and the mean and standard error of the non-classified vegetation contrast layer. We also estimated the edge length (i.e. the perimeter of the property). We used PCA to summarize edge characteristics (edge length, vegetation contrast and land cover types), followed by a hierarchical cluster analysis (complete linkage) using the first two dimensions of the PCA. Hierarchical clustering identified three edge types, with edges defined as permeable (i.e. mostly vegetated), semi-permeable and low permeability (mostly impervious surfaces), based on the degree of contrast between land cover types.

### Statistical analyses

All statistical analyses were conducted with R Version 4.03 [97]. We used generalized linear models (GLMs) with a binomial distribution and a log link function to assess the yard- and landscape-level features associated with the probability of nymph presence in yards, for each of the tick species collected. Small numbers of ticks precluded analyses based on density. Yard- and landscape-level features were initially explored in separate models, including separate landscape-level models for each buffer radius size around yards. The Akaike information criterion (AIC) was used to identify the most parsimonious model explaining variation in the presence of each tick species in yards from all possible combinations of explanatory variables (model selection using the ‘dredge’ function in the R package MuMIn) [98]. Variables significant in the yard- and landscape-level models were included in the global model. To account for model selection uncertainty, multimodel inference was used to quantitatively rank the best fit models, where models with an AIC difference (∆AIC) < 2 were designated as having similar support to the best model. Odds ratios (ORs) and their 95% confidence intervals (CIs) were calculated from model-averaged coefficients for each explanatory variable.

## Results

### Residential yard surveys

From April to September in 2018, 2019 and 2021, we conducted door-to-door recruitment at a total of 1988 houses. We were unable to speak with residents at 56% of these houses (i.e. either householders were not home at the time or did not open the door). Receptivity to recruitment was high among those who did open the door; 72% of residents approached were willing to participate in the study, with an average (± standard deviation [SD]) of 29 (± 1.5) houses participating in two of the years, and eight houses participating in all 3 years. A total of 529 unique yards were surveyed for ticks across SI, and each individual yard was only sampled once per season. We observed considerable variation in the presence of yard features among sites, with fully enclosed fencing being the most common feature, followed by water sources (e.g. swimming pools) and vegetable or flower gardens (Table 2).

**Table 2:** Frequency of residential yard features and yard feature association with the presence of ticks (*Amblyomma americanum*, *Haemaphysalis longicornis* or *Ixodes scapularis*)

Yard feature	Number (%) of residential yards with the feature	Number (%) of yards with the feature present from which ticks were collected
Fully enclosed fencing	302 (68)	71 (24)
Water source^a^	142 (54)	36 (26)
Vegetable or flower garden	274 (52)	84 (31)
Trashcan in yard^a^	114 (45)	33 (29)
Log or brush pile	144 (27)	73 (51)
Outdoor seating in lawn	133 (25)	44 (34)
Woodchips or gravel	130 (25)	45 (36)
Bird feeder	83(16)	26 (32)
Children’s play equipment	86 (16)	24 (29)
Food or shelter for feral cats^a^	32 (12)	17 (53)
Compost bin^a^	25 (10)	19 (37)
Chicken coop	6 (2)	0 (data not available)

^a^Sampled in 2021 only

Most yards contained some form of fencing (84%). Most houses had only a fully enclosed backyard (68%), followed by partial fencing (32%); only a few houses had a fully enclosed front yard and backyard (10%). For the analyses, we combined yards that had both the front yards and backyards completely fenced with those having only the backyard completely fenced into a “fully enclosed fencing” category (68%). Fence types included chain link, wooden picket, full panel, farm (broadly spaced horizontal wooden slats) and aluminum fences, and ranged from 0.3 to 2.5 m (mean: 1.60 m, SD: 0.35 m) in height. We categorized the fences into three height categories for analysis based on an assumed effect on deer movement [99]: no fence; non-deer-proof fence (< 1.8 m high); and deer-proof fence (> 1.8 m high).

**Fig. 2:**
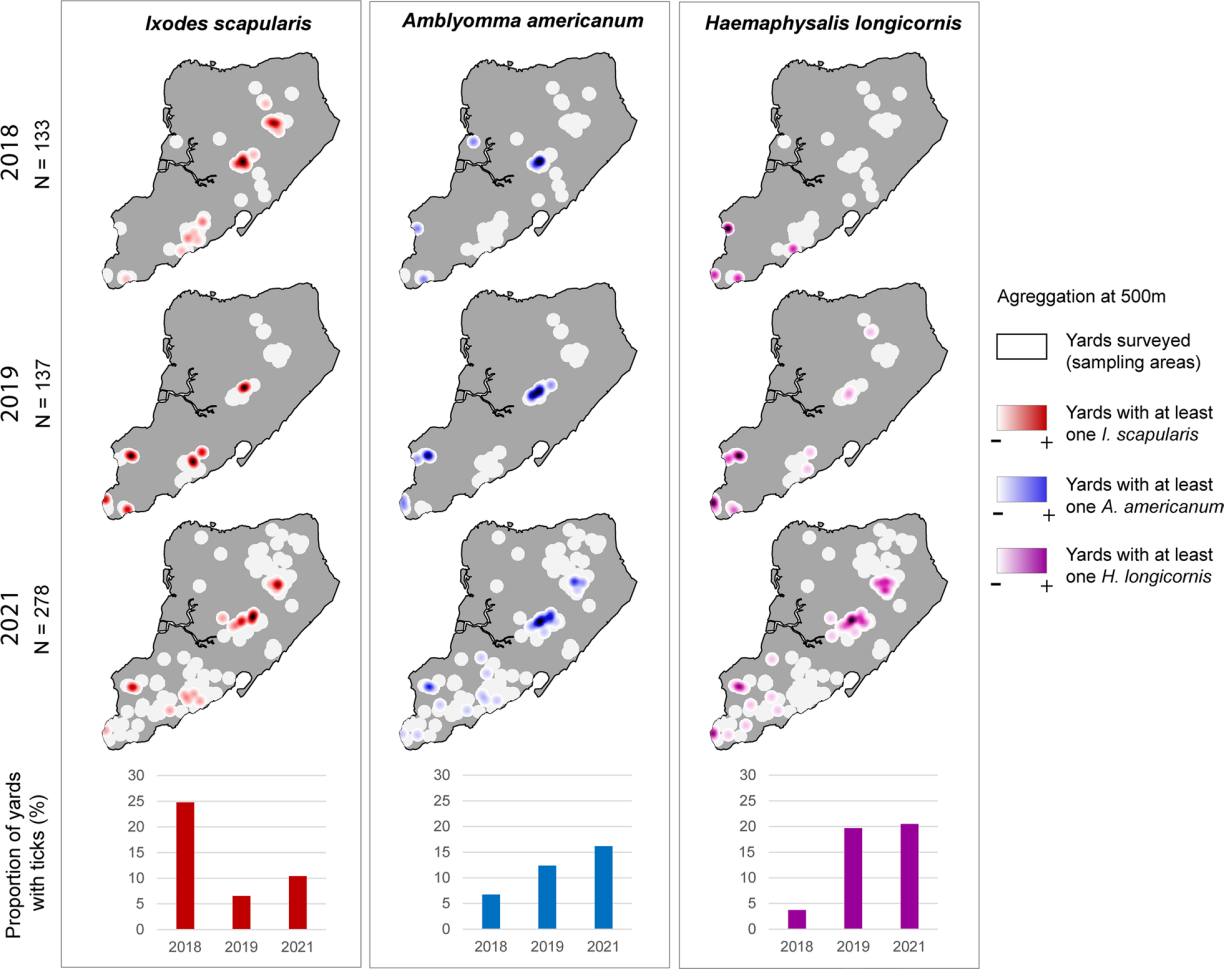
Kernel density estimate heatmaps of yards positive for each tick species across Staten Island over 3 field seasons (May—July)

The proportion of yards containing at least one tick of any species was consistently approximately 30% (range: 29–35%) over the 3 years (Fig. 2). However, the prevalence of each of the tick species in yards and their spatial distribution varied across years, with *I. scapularis* dominating the urban tick community in 2018 (35% houses), and *H. longicornis* most frequently observed in 2019 and 2021 (25% and 26% of houses, respectively; Fig. 2).

### Property edges: classification and relationship to block-type levels

The most common yards were those with permeable edges (*n* = 266), followed by semi-permeable (*n* = 195) and low permeability (*n* = 66) edge types. The distribution of edges varied across ecological neighborhoods, with yards in the northernmost sites (e.g. Clove Lakes) being dominated by impermeable edge types and those in the mid-island being largely semi-permeable and permeable.

### Land cover and landscape metric multivariate classification of urban yards

The proportion of land cover types was relatively consistent across buffer radii, with impervious surface, high and low canopy categories each comprising 20–30% of land cover classes (Table 3).

**Table 3:** Proportion of land cover classes in buffer radii around yards sampled

Land cover class	Buffer size (radius)			
	25 m	50 m	100 m	200 m
High canopy	0.22 (0.18)	0.24 (0.18)	0.30 (0.18)	0.35 (0.18)
Low canopy	0.29 (0.10)	0.28 (0.08)	0.27 (0.07)	0.26 (0.06)
Shrub	0.05 (0.02)	0.05 (0.01)	0.05 (0.01)	0.04 (0.01)
Grass	0.08 (0.05)	0.08 (0.04)	0.07 (0.03)	0.07 (0.03)
Impervious surface	0.35 (0.15)	0.33 (0.14)	0.30 (0.13)	0.27 (0.13)
Barren soil	0.008 (0.02)	0.01 (0.02)	0.007 (0.01)	0.006 (0.01)

Data in table are presented as the mean (standard deviation)

**Fig. 3:**
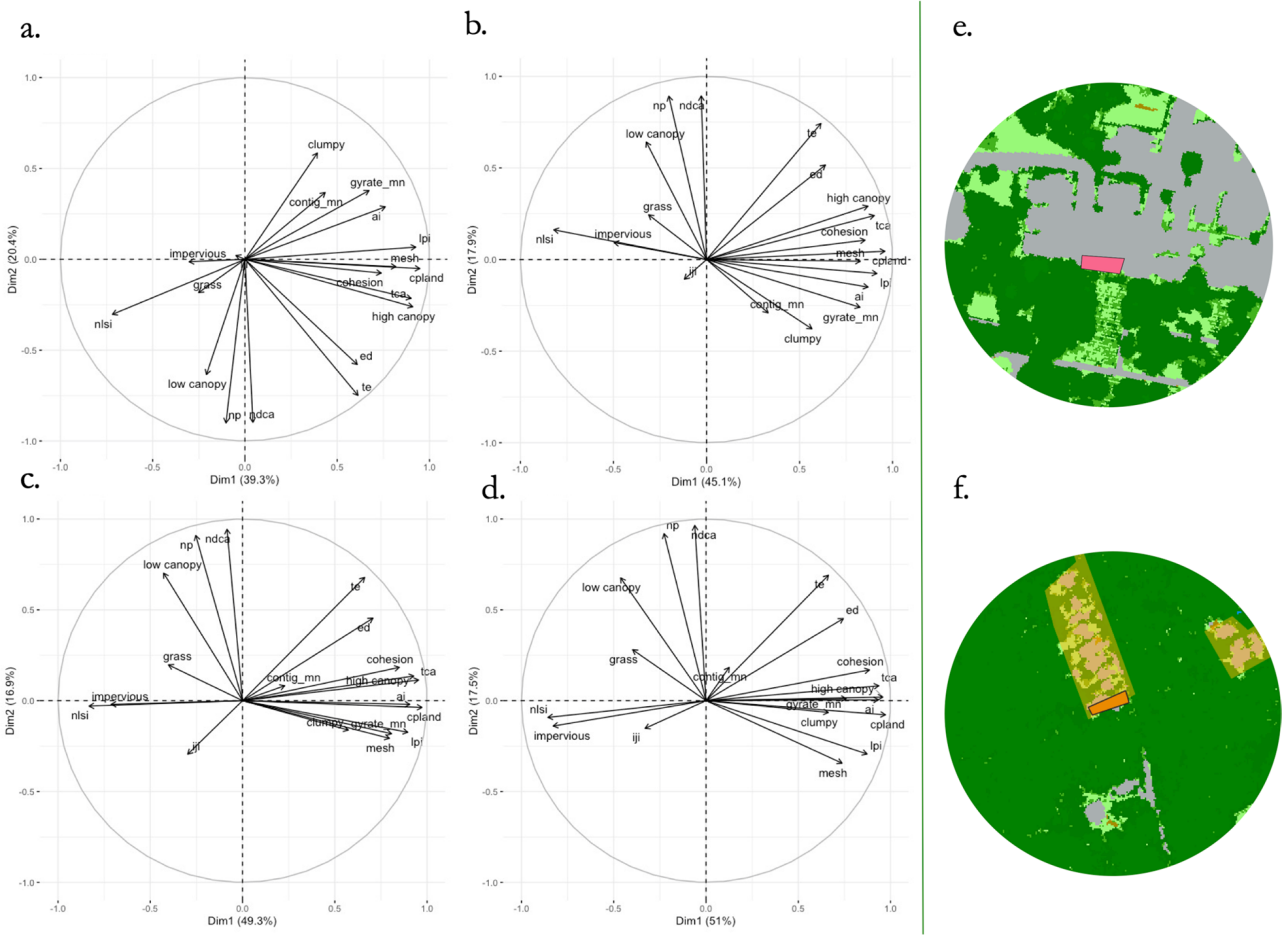
Biplot of principal component analysis (PCA) for landscape metrics and land cover in 25-m (**a**), 50-m (**b**), 100-m (**c**) and 200-m (**d**) buffers around residential yards, and examples of yards with low (**e**) and high (**f**) PC1 loadings. Abbreviations for landscape metrics: Cohesion, patch cohesion index; enn_mn, mean of Euclidean nearest neighbor; np, number of patches; clumpy, clumpiness index; nlsi, normalized landscape shape index; ai., aggregation index; iji, interspersion and juxtaposition index; mesh, effective mesh size; te, total edge; ed, edge density; lpi, largest patch index; gyrate_mn, mean radius of gyration; contig_mn, mean of contiguity index; tca, total core area; cpland, core area percentage of landscape; ndca, number of disjunct core areas. Abbreviations for landcover classes: Grass, area of grass; low canopy, area of low canopy; high canopy, area of high canopy; impervious, area of impervious surface

For the landscape metric PCA analysis, which included the 16 class-level landscape metrics, and areas of grass, canopy and impervious surface cover, the two first components combined explained between 56% and 70% of variation, with increasing length of the radii length representing greater proportions of variation. For all radii, increasing values of PC1 (the first dimension of the PCA analysis) were positively correlated with area of high canopy cover and high values for aggregation and core area metrics (Fig. 3) and negatively associated with impervious cover. Thus, yards with high PC1 loadings contained large, compact and connected patches of canopy within the surrounding buffer area (Fig. 3). Increasing values of PC2 (the second dimension of the PCA analysis) were positively correlated with number of disjunct core areas, number of patches and grass and low canopy cover (Additional file 1: Table S1), indicative of smaller, disaggregated patches of lower canopy cover classes, for radii of 50, 100 and 200 m, respectively. The same features correlated with PC2 for radius of 25 m, although the sign was reversed (i.e. negative rather than positive association with PC2).

### Yard- and landscape-level features associated with tick presence

The probability of detecting each of the three tick species was associated with different yard- and landscape-level features and was in all cases best explained by models containing parameters from both yard and landscape scales. All species showed significant associations with year, with the probability of finding *I. scapularis* decreasing over the sampling period, and the probabilities of finding *A. americanum* and *H. longicornis* increasing over the same period by an average of three- and ninefold, respectively.

In the yard-level-only model, log and brush piles significantly increased the odds of finding all three tick species (Additional file 1: Table S2). This effect was particularly strong for *H. longicornis* and *A. americanum,* which were associated with 3.6- and 4-fold increases in the odds of tick presence, respectively. Woodchips and gravel at the edge of yard properties increased the probability of finding *A. americanum* (OR: 2.3, 95% CI: 1.2–4.3; *P* = 0.01), but not for finding either of the other species. Full fencing of any kind around the yards decreased the odds of *I. scapularis* being present (OR: 0.43, 95% CI: 0.22–0.87; *P* = 0.01), but had no effect on *A. americanum* (*P* = 0.07) or *H. longicornis* (*P* = 0.10). The permeability of the property edge impacted the probability of finding *A. americanum* but not *H. longicornis* or *I. scapularis*, and was only significant for a permeable/semi-permeable edge contrast (OR: 0.47, 95% CI: 0.23–0.96; *P *= 0.04).

In the landscape-level-only model, the distribution of ticks was best predicted by landscape metrics at different scales. PC1 calculated at the 100-m buffer best predicted *I. scapularis* distribution (OR: 1.45, 95% CI: 1.3, 1.7; *P* < 0.001); PC1 at the 50-m buffer best predicted *A. americanum* (OR: 1.2, 95% CI: 1.0–1.4; *P* = 0.01); and PC1 was not significant at any scale for *H. longicornis*. PC2 was not significant in any models (Additional file 1: Table S3).

In the global model, *I. scapularis* presence was best predicted by PC1 at the 100-m buffer, the presence of log or brush piles and sampling year. Log and brush piles and woodchips remained the strongest predictors of *A. americanum* presence in yards, followed by PC1 at the 50-m buffer. Additionally, the density of *A. americanum* nymphs per 100 m in nearby parks increased the odds of detecting *A. americanum* ticks in yards. Landscape metrics had no effect on the odds of detecting *H. longicornis*, which was associated with the presence of log and brush piles and the density of conspecific nymphs in the nearest park. Sampling year had the strongest effect on *H. longicornis*, with the odds of finding them in 2021 ninefold greater than in 2018. For all species, the presence of fully enclosed fencing was retained in the models and had a negative association; however the effect was no longer significant when the landscape-level metrics were included (Additional file 1: Table S2).

## Discussion

Tick-borne diseases present an increasing threat in urban areas, where high human density can intensify human exposure to ticks given the presence of a suitable tick habitat and competent host niches. In the present study, we highlight the considerable risk of tick exposure in residential yards on SI and demonstrate that the dynamics of three tick vector species in a highly fragmented urban environment are determined by both yard- and landscape-level features. In particular, we found that the distributions of the Lyme disease spirochetes vector *I. scapularis* and *A. americanum* were largely determined by yard- and landscape- level factors, whereas the distribution of *H. longicornis* was only impacted by yard-level features but not landscape level factors assessed in this study.

Tick survival depends on the local abiotic and biotic conditions [5, 38, 53, 54]. The finding that *I. scapularis* presence is most strongly associated with the amount of large, well-connected patches of canopy cover in the surrounding landscape is in line with previous work linking the species to forest habitat and connectivity [46, 75, 100–104]. Underlying this relationship is the sensitivity of *I. scapularis* to desiccation, which may constrain the tick to patches of canopy cover in urban areas, outside of which high impervious surface cover dramatically increases local temperatures and reduces saturation deficit [105, 106]. Specifically on SI, VanAcker et al. [46] showed that the percentage of bare soil, impervious surface, water and grass in a buffer of 100 m around parks reduced the density of *I. scapularis* nymphs in parks. Both *A. americanum* and *H. longicornis* are more tolerant to desiccation and heat stress than *I. scapularis*, allowing them to persist in a range of habitats. In particular, *H. longicornis* can withstand temperatures up to 40 °C and severe dehydrating conditions under laboratory conditions [107, 108]. Differences in the distributions of *A. americanum* and *H. longicornis* may be explained by these differences in environmental tolerances as well as the time since their invasion.

At the yard level, log and brush piles were consistently associated with the presence of all tick species. Log and brush piles may act as thermal refugia for ticks, allowing them to persist and to quest near open lawns where they would otherwise desiccate, and may also act as habitat for small-bodied hosts, such as mice, and dens for meso-mammals, such as raccoons [109, 110]. Brush piles increase overwinter survival of white-footed mice [111], which act as important hosts for *I. scapularis* larvae, and the distribution of which may determine where fed larvae are deposited and emerge the following spring as nymphs. Landscaping tick control measures (e.g. clearing brush piles) have been found to increase the risk of *I. scapularis-*associated disease, potentially by increasing exposure to ticks [100], but to have no effect on Lyme disease cases [112]. These different findings may reflect the discordant scales and metrics at which the two studies were conducted, the former being a meta-analysis of several studies that aggregated log and brush clearing into a property management risk category, and the latter being a neighborhood-matched case–control study. Our finding of a higher probability of nymph presence with log and brush piles may or may not lead to increased human tick exposure and disease depending on human exposure behavior. Further work elucidating human behavior in similar urban settings would provide insights into the relative roles of the natural and human components of urban TBD risk.

Landscape connectivity linked to host movement is an important determinant of tick distribution. The adult stages of all tick species in the present study are dependent on white-tailed deer as the primary reproductive stage host [11] and for movement through landscapes [46]. However, *A. americanum* and particularly *H. longicornis* are also associated with mesomammal hosts, such as raccoons and opossums [27, 107, 113–115]. Adult-stage *A. americanum* and *H. longicornis* have been found to exhibit generalist feeding behavior, feeding on a range of livestock, birds and small mammal species [107, 116], which would allow them to feed on hosts less limited by landscape structure and fences than deer (e.g. squirrels and racoons [117]). The association between fencing and nymphal *I. scapularis,* but not *A. americanum* or *H. longicornis*, may thus reflect differences in proportional host use of adults, which determines to some extent subsequent larval and nymphal distributions, between the three species.

Differences in host use and differences in the impact of the landscape on host movement (i.e. its functional connectivity [119]) may also explain the positive association between *I. scapularis* and *A. americanum* in residential yards and canopy cover and connectivity in surrounding yards, but not *H. longicornis.* Previous work in NYC found considerably greater burdens of immature stages of *A. americanum* and *H. longicornis* on raccoons than *I. scapularis* [115]. Additionally, opossums were found to have highest infestation prevalence and intensity of immature *H. longicornis* than either *I. scapularis* or *A. americanum*, providing some support for differences in host use. However, as we did not conduct mammal trapping in yards to assess ticks on hosts for this study, we can only speculate on host movement being the mechanism for the observed patterns.

It is important to note that while we identify risk factors for tick presence in residential yards, reducing the acarological hazard alone does not necessarily result in a concomitant decrease in the incidence of tick-borne disease. In a recent experiment conducted over 4 years in a residential neighborhood in NYC, two tick control methods effectively reduced the number of questing ticks, ticks on rodents and TBD in pets, but they had no discernable effect on the incidence of human TBDs [120]. While small sample size per neighborhood, relatively few TBD cases over the study period and variation in human preventative behaviors may have all played a role in decoupling tick abundance from human incidence [120], the present study highlights the need to better understand the coupling between tick distribution and human exposure behaviors across human-dominated landscapes in order to evaluate where exposure is most frequently occurring.

Finally, we identified a potential invasion front for *H. longicornis*, and potentially for *A. americanum*, which increased ninefold and threefold, respectively, from 2018 to 2021. The concomitant decrease in *I. scapularis* over the study period, and similar observations in several studies in the region raises the question of whether environmental conditions may be changing that favor these expanding tick species over *I. scapularis*. More speculatively, *H. longicornis* and *A. americanum* may be currently displacing *I. scapularis*, although an ecological mediating mechanism has not been identified. Variation in climate may be expected to impact tick populations; however, in the present study mean summer temperatures were similar across the sampling years [121, 122]. A decline in *I. scapularis* relative abundance may also simply be the result of yearly population variation resulting from the tick’s 2- to 3-year life-cycle. Understanding the environmental factors associated with the distribution of these expanding tick populations, at appropriate spatial scales, is a critical first step towards guiding future policy regarding tick surveillance and management recommendations for individuals in high-risk areas.

## Conclusions

Proximity to parks and the amount and aggregation of forest canopy immediately (50–100 m) surrounding residential properties is a key risk factor for finding ticks in yards, particularly the Lyme disease spirochetes vector, *I. scapularis*. However, complete fencing and removal of log and brush piles can mitigate landscape-mediated effects on the tick hazard by impeding host movement through yards and decreasing the amount of suitable habitat available for wildlife hosts and ticks.

## Supplementary Information

**Additional file 1: Table S1.** PCA loadings for land cover and landscape metrics in buffer radii around residential yards. **Table S2.** Model-average odds ratios and 95% confidence limits of covariates in yard-only generalized linear models of tick presence in yards. **Table S3.** Best fit models for full models (including all features) of tick presence in yards. **Figure S1.** Dragging locations for tick sampling in a residential yard. Numbers correspond to transects, and letters denote 10-m sections of each transect.

## Data Availability

The datasets used and/or analysed during the current study are available from the corresponding author on reasonable request.

## Abbreviations

NYC: New York City
PCA: Principal component analysis
SI: Staten Island
TBD: Tick-borne disease
