# Supplementary material for: Risk of tick-borne pathogen spillover into urban yards in New York City
Source: Parasit Vectors. 2022 Aug 10;15:288. doi: 10.1186/s13071-022-05416-2 (PMC9365221; doi:10.1186/s13071-022-05416-2)
Supplement: Supplementary file 1 — Additional file 1: Table S1. PCA loadings for land cover and landscape metrics in buffer radii around residential yards. Table S2. Model-average odds ratios and 95% confidence limits of covariates in yard-only generalized linear models of tick presence in yards. Table S3. Best fit models for full models (including all features) of tick presence in yards. Figure S1. Dragging locations for tick sampling in a residential yard. Numbers correspond to transects, and letters denote 10-m sections of each transect. [file 13071_2022_5416_MOESM1_ESM.docx]

Risk of tick-borne pathogen spillover into urban yards in New York City:

Nichar Gregory^1^, Pilar Maria Fernandez^1,2,3^, Maria Diuk-Wasser^1^

^1^Department of Ecology, Evolution and Environmental Biology, Columbia University, New York 10027

^2^Earth Institute, Columbia University, New York, NY, ^3^Paul G. Allen School for Global Health, Washington State University, Pullman, WA

**Supplementary Information:**

**Additional file 1**

**Table S1. PCA loadings for landcover and landscape metrics in buffer radii around residential yards.**

|  | 25m | | 50m | | 100m | | 200m | |
| --- | --- | --- | --- | --- | --- | --- | --- | --- |
|  | PC1 | PC2 | PC1 | PC2 | PC1 | PC2 | PC1 | PC2 |
| Area grass | -0.09 | -0.09 | -0.11 | 0.13 | -0.13 | 0.11 | -0.13 | 0.15 |
| Area low canopy | -0.08 | -0.32 | -0.11 | 0.35 | -0.14 | 0.39 | -0.15 | 0.37 |
| Area high canopy | 0.33 | -0.13 | 0.30 | 0.16 | 0.31 | 0.06 | 0.31 | 0.01 |
| Area impervious | -0.11 | -0.01 | -0.17 | 0.05 | -0.23 | -0.01 | -0.27 | -0.08 |
| AI | 0.28 | 0.15 | 0.30 | -0.08 | 0.30 | -0.01 | 0.30 | 0.00 |
| CLUMPY | 0.14 | 0.30 | 0.19 | -0.20 | 0.19 | -0.09 | 0.21 | -0.04 |
| COHESION | 0.27 | -0.04 | 0.29 | 0.06 | 0.28 | 0.10 | 0.28 | 0.09 |
| CONTIG_MN | 0.16 | 0.19 | 0.11 | -0.16 | 0.07 | 0.05 | 0.04 | 0.10 |
| CPLAND | 0.35 | -0.03 | 0.33 | 0.02 | 0.32 | -0.02 | 0.31 | -0.04 |
| ED | 0.22 | -0.29 | 0.22 | 0.28 | 0.23 | 0.25 | 0.24 | 0.25 |
| GYRATE_MN | 0.25 | 0.19 | 0.28 | -0.14 | 0.26 | -0.10 | 0.24 | 0.01 |
| IJI | -0.02 | 0.01 | -0.04 | -0.06 | -0.10 | -0.16 | -0.11 | -0.08 |
| LPI | 0.34 | 0.03 | 0.31 | -0.04 | 0.29 | -0.10 | 0.28 | -0.16 |
| MESH | 0.30 | -0.02 | 0.28 | 0.00 | 0.26 | -0.12 | 0.24 | -0.19 |
| NDCA | 0.02 | -0.46 | -0.01 | 0.48 | -0.03 | 0.53 | -0.02 | 0.53 |
| NLSI | -0.26 | -0.15 | -0.28 | 0.09 | -0.27 | -0.02 | -0.28 | -0.05 |
| NP | -0.04 | -0.46 | -0.07 | 0.48 | -0.08 | 0.51 | -0.07 | 0.50 |
| TCA | 0.33 | -0.11 | 0.31 | 0.13 | 0.30 | 0.08 | 0.30 | 0.04 |
| TE | 0.22 | -0.38 | 0.21 | 0.40 | 0.22 | 0.38 | 0.21 | 0.38 |

**Table S2. Model-average odds ratios and 95% confidence limits of covariates in yard-only generalized linear models of tick presence in yards.**

| **Species** | **Odds ratio** | **Lower CI** | **Upper CI** | ***p*** |
| --- | --- | --- | --- | --- |
| **Covariate** |  |  |  |  |
| ***Ixodes scapularis*** | | | | |
| Distance to nearest park | 0.99 | 1.90 | 130 | 0.00*** |
| Epidemiological week | 1.00 | 0.99 | 1.00 | 0.00*** |
| Log or brush pile | 2.61 | 1.40 | 4.84 | 0.00*** |
| Fencing | 0.43 | 0.22 | 0.87 | 0.02** |
| Year 2019 | 0.18 | 0.08 | 0.43 | 0.00*** |
| 2021 | 0.19 | 0.09 | 0.41 | 0.00*** |
| Vegetable or flower garden | 0.89 | 0.48 | 1.65 | 0.85 |
| ***Haemaphysalis longicornis*** | | | | |
| Epidemiological week | 1.00 | 0.99 | 1.00 | 0.51 |
| Log or brush pile | 3.60 | 1.69 | 5.82 | <0.001*** |
| Fencing | 0.62 | 0.35 | 1.11 | 0.10 |
| Year 2019 | 10.00 | 2.81 | 35.65 | <0.001*** |
| 2021 | 8.03 | 2.31 | 27.93 | 0.01** |
| Vegetable or flower garden | 1.32 | 0.73 | 2.40 | 0.36 |
| Area impervious surface | 1.00 | 1.00 | 1.00 | 0.70 |
| Edge type: semi-permeable | 0.97 | 0.53 | 1.79 | 0.90 |
| low permeable | 0.37 | 0.08 | 1.69 | 0.20 |
| ***Amblyomma americanum*** |  |  |  |  |
| Epidemiological week | 1.00 | 0.99 | 1.00 | <0.001*** |
| Log or brush pile | 4.00 | 1.90 | 6.76 | <0.001*** |
| Fencing | 0.56 | 0.30 | 1.05 | 0.07 |
| Edge type: semi-permeable | 0.47 | 0.23 | 0.96 | 0.04 |
| low permeable | 0.30 | 0.07 | 1.37 | 0.12 |
| Woodchips or gravel | 2.30 | 1.20 | 4.30 | 0.01* |
| Year 2019 | 3.55 | 1.27 | 9.96 | 0.02* |
| 2021 | 2.56 | 0.94 | 6.95 | 0.07 |
| Area impervious surface | 1.00 | 1.00 | 1.00 | 0.60 |
| Vegetable or flower garden | 1.00 | 0.99 | 1.00 | 0.73 |
| Distance to nearest park | 3.58 | 1.90 | 6.76 | 0.88 |

*Models ranked by increasing AIC, and models with weights <0.001 have been removed.*

**Table S3. Best fit models for full models (including all features) of tick presence in yards.**

| **Species** | **Odds ratio** | **Lower CI** | **Upper CI** | ***p*** |
| --- | --- | --- | --- | --- |
| **Covariate** |  |  |  |  |
| ***Ixodes scapularis*** | | | | |
| PC1 at 100m | 1.42 | 1.24 | 1.62 | <0.001*** |
| Distance to nearest park | 1.00 | 0.99 | 1.00 | 0.11 |
| Epidemiological week | 1.00 | 0.99 | 1.00 | 0.001* |
| Log or brush pile | 2.28 | 1.19 | 4.35 | 0.01* |
| Fencing | 0.56 | 0.30 | 1.07 | 0.30 |
| Year 2019 | 0.23 | 0.10 | 0.54 | <0.001*** |
| 2021 | 0.23 | 0.10 | 0.51 | <0.001*** |
| Vegetable or flower garden | 1.15 | 0.61 | 2.18 | 0.88 |
| ***Haemaphysalis longicornis*** | | | | |
| Epidemiological week | 1.00 | 0.99 | 1.00 | 0.51 |
| Log or brush pile | 3.13 | 1.69 | 5.82 | 0.00*** |
| Fencing | 0.62 | 0.35 | 1.11 |  |
| Year 2019 | 10.00 | 2.81 | 35.65 | 0.28 |
| 2021 | 9.00 | 2.31 | 27.93 | 0.00*** |
| Vegetable or flower garden | 1.32 | 0.73 | 2.40 | 0.00*** |
| Area impervious surface | 1.00 | 1.00 | 1.00 | 0.77 |
| Edge type: semi-permeable | 0.97 | 0.53 | 1.79 | 0.90 |
| low permeable | 0.37 | 0.08 | 1.69 | 0.97 |
| ***Amblyomma americanum*** |  |  |  |  |
| *A. americanum* density in nearest park | 1.03 | 1.02 | 1.05 | 0.001** |
| PC1 at 100m | 1.20 | 1.05 | 1.39 | 0.01** |
| Distance to nearest park | 1.00 | 1.00 | 1.01 | 0.44 |
| Epidemiological week | 1.00 | 0.99 | 1.00 | 0.05* |
| Log or brush pile | 4.12 | 2.01 | 8.44 | <0.001*** |
| Fencing | 0.50 | 0.24 | 1.04 | 0.18 |
| Woodchips or gravel | 2.48 | 1.17 | 5.23 | 0.17 |
| Year 2019 | 3.28 | 1.00 | 10.76 | 0.20 |
| 2021 | 3.20 | 1.03 | 9.92 | 0.20 |
| Edge type: semi-permeable | 0.50 | 0.22 | 1.16 | 0.54 |
| low permeability | 0.32 | 0.04 | 2.94 | 0.63 |

.


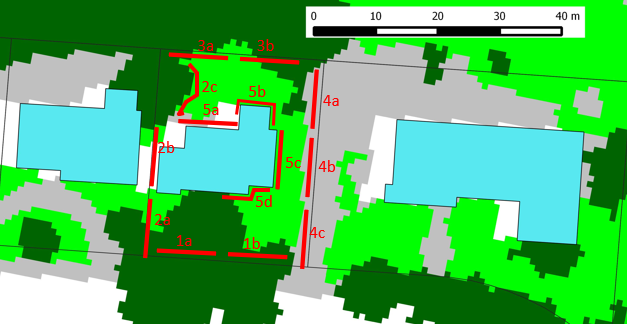


**Figure S1. Dragging locations for tick sampling in a residential yard.** Numbers correspond to transects, and letters denote 10m sections of each transect.
